# Poor acclimation to experimental field drought in subalpine forest tree seedlings

**DOI:** 10.1093/aobpla/plab077

**Published:** 2021-12-17

**Authors:** Alex Goke, Patrick H Martin

**Affiliations:** 1 Department of Botany, University of Wisconsin-Madison, Madison, WI 53706, USA; 2 Department of Biological Sciences, University of Denver, Denver, CO 80210, USA

**Keywords:** *Abies lasiocarpa*, allometry, drought, gas exchange, monsoon, *Picea engelmannii*, seedling, subalpine

## Abstract

The ability of tree species to acclimate and tolerate projected increases in drought frequency and intensity has fundamental implications for future forest dynamics with climate change. Inquiries to date on the drought tolerance capacities of tree species, however, have focused almost exclusively on mature trees with scant in *situ* work on seedlings, despite the central role that regeneration dynamics play in forest responses to changing conditions. We subjected naturally established seedlings of co-dominant subalpine conifer species (*Abies lasiocarpa* and *Picea engelmannii*) in the southern Rocky Mountains to 2 years of *in situ* summer precipitation exclusion, simulating summer drought conditions similar to a failure of the North American monsoon. We compared the morphological and physiological responses of seedlings growing in drought vs. ambient conditions to assess the relative changes in drought tolerance traits as a function of seedling size. Drought treatments had a marked impact on soil moisture: volumetric water content averaged ≈5–8 % in drought treatments and ≈8–12 % in ambient controls. We detected no significant shifts in morphology (e.g. root biomass, leaf:stem area ratio) in response to drought for either species, but net photosynthesis in drought treatments was 78 % lower for spruce and 37 % lower for fir. Greater stomatal control associated with increasing stem diameter conferred greater water use efficiencies in larger seedlings in both species but was not significantly different between drought and ambient conditions, suggesting an overall lack of responsivity to water stress and a prioritization of carbon gain over investment in drought mitigation traits. These results indicate a canonization of traits that, while useful for early seedling establishment, may portend substantial vulnerability of subalpine seedling populations to prolonged or recurrent droughts, especially for spruce.

## Teaser

Knowledge of species’ ability to tolerate projected increases in drought is necessary to anticipate future forest dynamics with climate change, but few studies have investigated the responses of tree seedlings to drought in the field. Subjecting naturally regenerating spruce and fir seedlings to drought in the subalpine Rocky Mountains, we observed a striking lack of morphological and physiological acclimation to drought, and a prioritization of carbon gain traits at the expense of drought mitigation and tolerance. These results indicate canonization of traits that, while useful for early seedling establishment, may indicate substantial vulnerability of seedling populations to prolonged or recurrent droughts.

## Introduction

Droughts are expected to increase in both frequency and intensity with ongoing climate change. Droughts linked to climate change have already caused widespread tree mortality across large areas of many forested regions, with deeply adverse impacts on landscape structure and function (Van Mantgem *et al.* 2009; Allen *et al.* 2010; Anderegg *et al.* 2013; Clark *et al.* 2016). These drought episodes are of particular concern for high-elevation forests in mountainous regions where climate change is accelerated relative to lower elevations (Beniston *et al.* 1997; IPCC 2014; Pepin *et al.* 2015; Dobrowski and Parks 2016) and resistance of forests to environmental shifts is strongly affected by competitive interactions (Buechling *et al.* 2017), divergent species responses (Carroll *et al.* 2017, 2021) and low phenotypic plasticity due to strong local adaptation to narrow bioclimatic envelopes (Valladares *et al.* 2007, 2014; Vitasse *et al.* 2013; Gugger *et al.* 2015). Recent progress in characterizing the underlying causes of drought-induced tree mortality has highlighted the value of plasticity in mitigating drought stress and reducing likelihood of mortality, especially in traits associated with water and carbon regulation strategies (Richter *et al.* 2012; Choat *et al.* 2018). However, there are few studies on how drought affects natural populations of tree seedlings in the field, despite the critical role seedlings play in forest dynamics, range shifts and the overall resilience of forests to ongoing climate change (Bell *et al.* 2014; Martínez-Vilalt and Lloret 2016; Brodersen *et al.* 2019; Copenhaver-Parry *et al.* 2020; Foster *et al.* 2020).

Relative to saplings and adults, which overall display strong synchronization to their environment (Martin and Canham 2020) and increased stress tolerance, conifer tree seedlings are more vulnerable to water stress given their shallow rooting depths and narrow carbon budgets (Grossnickle 2005; Bansal and Germino 2008, 2010; Niinemets 2010; Gill *et al.* 2015). These limitations are most pronounced in newly emerged germinants where trade-offs between investments in leaf development for photosynthesis and growth and structural stress mitigation traits are often observed in conjunction with high mortality rates (Green 2005; Reinhardt *et al.* 2015; Lazarus *et al.* 2018; Augustine and Reinhardt 2019). Instead of prioritizing allocation to photosynthetic development, seedlings could invest energy in water-stress mitigation strategies such as increased carbon allocation to below-ground structures to support water uptake, adjustment of the leaf:sapwood area ratio to promote more suitable whole-plant water relations or tighter control of stomatal conductance towards increased photosynthetic water use efficiency (Choat *et al.* 2018). However, many studies have demonstrated a striking lack of morphological changes in small conifer seedlings—particularly first-year germinants—in response to water deficits (e.g. Aranda *et al.* 2010; Schall *et al.* 2012; Augustine and Reinhardt 2019). Indeed, conifer seedlings under water stress may respond with even greater investments in leaf development at the expense of root mass (e.g. Kiorapostolou *et al.* 2018), suggesting allocation hierarchies prioritizing carbon gain over drought mitigation traits are canalized in these seedlings—potentially at the risk of less favourable water status (e.g. greater whole-plant transpiration resulting from increased leaf area). While trees are generally recognized as having increased stress tolerance and morphological acclimation capacities as they grow larger (Niinemets 2010), how and when small seedlings begin to display more conservative physiological controls and alter carbon allocation patterns to develop water-stress mitigation traits more reflective of conspecific adults is largely unknown.

In the present study, we investigated allometry and gas exchange characteristics of naturally regenerated established seedlings of Engelmann spruce (*Picea engelmanii* Parry ex Engelm.) and subalpine fir (*Abies lasiocarpa* (Hooker) Nuttall) over two growing seasons in response to late-season simulated drought in the Front Range of Colorado, USA. Spruce and fir are the dominant tree species across a wide elevational and mesoclimate band in subalpine forests in the Rocky Mountains and have experienced dramatic increases in tree mortality in recent decades associated with increasing moisture stress (Bigler *et al.* 2007; Smith *et al.* 2015; Andrus *et al.* 2021). Increasing temperatures and associated declines in snowpack and earlier snowmelt have lengthened the period of water deficits across western mountains, and summer precipitation in the southern Rocky Mountains is declining due to the systematic weakening of the North American monsoon (Mote *et al.* 2005; Hu *et al.* 2010; Cook and Seager 2013; Pascale *et al.* 2017). These changes in the region’s moisture regime could impact spruce and fir differently given their contrasting life-history strategies. Relative to spruce, fir has greater seedling rooting depth (Day 1964) and slower growth rates (Day 1964; Antos *et al.* 2000), but higher rates of net photosynthesis at lower levels of light saturation (Knapp and Smith 1982), and earlier stomatal closure at the onset of environmental stress, enhancing water use efficiency in stressful environments (Knapp and Smith 1981; Brodersen *et al.* 2006). Collectively, these factors are hypothesized to lead to the high abundance of fir often observed in subalpine forest understories, while more rapid growth rates and greater longevity in spruce facilitate its overstory co-dominance, with seedlings that capitalize on higher light levels in canopy gaps (Shea 1985; Veblen 1986; Andrus *et al.* 2018).

The goals of this study were to evaluate monsoon failure-type drought response strategies of high-elevation conifer seedlings, and to compare patterns of biomass allocation and gas exchange between small seedlings and adult-sized individuals. Though prior studies did not directly evaluate the *in situ* effects of drought on co-occurring Engelmann spruce and subalpine fir seedlings, a body of research suggests species-specific differences in seedling responses to drought (Conlisk *et al.* 2018; Lett and Dorrepaal 2018; Brodersen *et al.* 2019). Based on this work, we predict (i) seedlings will acclimate to drought through morphological and physiological changes (e.g. allocate more growth to roots to alleviate water stress or reduce leaf area to limit transpiration losses), and that spruce—due to an inherently higher growth rate and delayed stomatal closure which could lead to more rapid desiccation—will exhibit greater morpho-physiological responses; (ii) due to slower growth and lower photosynthetic rates, fir will display superior overall drought tolerance than spruce via greater sustained rates of photosynthesis and water use efficiency under induced drought; and (iii) smaller individuals of both species will generally exhibit weaker morphological changes and lower photosynthetic water use efficiencies than larger individuals due to narrower carbon budgets and less-regulated stomatal behaviour. If accurate, these predictions suggest further recruitment failure for spruce during growing-season droughts, potentially disrupting successional dynamics of these two species. Alternatively, since growth rates and available carbon in seedlings of both species are low, neither species may sufficiently shift its morphology and physiology, leading to drastic reductions in photosynthesis and transpiration that could result in general recruitment failure of these dominant subalpine species under recurring future drought.

## Materials and Methods

### Site description

This study was conducted in 2018 and 2019 at the University of Denver High Altitude Laboratory near Mt. Evans, CO, USA (39.66°N, 105.59°W). At 3230 m in elevation, the northeastern-facing site is co-dominated by mature subalpine fir and Engelmann spruce with a few dispersed individuals of lodgepole pine (*Pinus contorta* Douglas), limber pine (*Pinus flexilis* E. James) and bristlecone pine (*Pinus aristata* Engelm). These site conditions are reflective of a compositional equilibrium characteristic of mature (>200-year-old) spruce–fir subalpine forest (Andrus *et al.* 2018). The patchy understory is composed primarily of ericaceous species (*Vaccinium* spp. and *Orthillia secunda* (L.) House) along with seedlings and saplings of fir and spruce, with fir occurring in notably greater proportions than spruce. Soils consist of mainly Leighcan family till substratum and Tonahutut-Ohman complex derived from igneous and metamorphic rock (NRCS 2020). No evidence of any recent disturbances (fire, blow-down, insect outbreak, etc.) was apparent at the site during the period of study. Mean annual temperature is 2.6 °C, and mean annual precipitation is 78 cm, most of which is snow but with notable rain input from summer monsoons occurring July to September (≈30 % of annual precipitation; NRCS SNOTEL 2020). Monsoon rains are particularly important as the study site is far enough from the climatic boundary between the arid west and the humid east typically located in Great Plains that even periods of high precipitation in the western Great Plains do not extend far enough west to increase moisture in the Front Range mountains of Colorado (Salley et al. 2016). Though total annual precipitation in the study region is projected remain within 5 % of historical levels in the next century, reductions in snowpack and monsoon circulation along with rising temperature are likely to exacerbate growing-season moisture deficits (Mote *et al.* 2005; Lukas *et al.* 2014). Mean summer temperatures (June–September) at the site during the study periods were similar (12.1 % higher in 2018 and 7.5 % higher in 2019) to recent averages (1999–2017, NRCS SNOTEL 2020; see Supporting Information—Table S1). Total winter precipitation (October–May) prior to the growing seasons in 2018 and 2019 was 24.7 % lower and 4.6 % higher than recent averages, respectively.

### Experimental design

Forty 1 × 1 m plots containing naturally regenerated Engelmann spruce and subalpine fir seedlings were established throughout the understory of the study site. Plots were selected for their approximate uniformity in litter composition, consistent microtopography (<15° slope) and herbaceous cover (<5 % area). Light availability within each plot was quantified using a hemispherical camera lens placed at seedling height (COOLPIX 900, Nikon, Tokyo, Japan) and expressed as % of potential direct and diffuse transmitted light based on latitude and topography using gap light analysing software (GLA v. 2.0, Cary Institute for Ecosystem Studies, Millbrook, NY, USA; Frazer *et al.* 1999). Each pair of plots (ambient and drought) was located >10 m from the nearest neighbouring plot pair, resulting in a total study area of ~3 ha.

Seedlings within each plot were marked and surveyed for height and caliper stem diameter at the root crown so that similar numbers and sizes of seedlings were present in all 40 plots. All seedlings were less than 20 cm in height and between 0.5 and 5 mm in stem diameter, representing a broad class of post-establishment seedlings residing in the understory **[see****Supporting Information—Fig. S1****]**. No more than 20 individual seedlings were contained in any plot to control for possible competitive effects. Half of the plots received a precipitation exclusion treatment (‘drought’) and half were used as paired control plots receiving ambient levels of precipitation (‘ambient’) and located immediately to the side or upslope of the treatment plots while maintaining a 0.5-m buffer to prevent additional precipitation accumulation in control plots from exclusion shelter run-off.

To impose drought treatments via precipitation exclusion, 20 passive rain deflection shelters were constructed, with one located above each drought plot. Using validated designs (Yahdjian and Sala 2002; Drought-Net 2018), the shelters were constructed to cover the 1-m^2^ plots (1 × 1 m) with roofs angled towards the downhill side of the shelters to drain water away from the target and control plots. The shelters were constructed to reduce precipitation by 100 %. Roofs were made of transparent polycarbonate roofing (Suntuf® Clear, Palram Americas, Kutztown, PA, USA) mounted 1 m above the soil surface on a frame constructed of PVC pipe. Shelters were in place above the plots from 15 July to 15 September in 2018 and 2019 to mimic summer monsoon failure, thereby creating a summer drought treatment for two consecutive growing seasons. Several prior studies have examined the effects of such shelters on the micro-environment and found air and soil temperatures to be minimally altered, with light transmission largely agreeable to manufacturer’s specifications (Fay *et al.* 2000; Yahdjian and Sala 2002; Heisler-White *et al.* 2008; Cherwin and Knapp 2012). Therefore, while systematic micro-environmental artefacts may be present, this shelter design has minimized potential light and temperature effects to a degree in which they are likely inconsequential relative to the broader range of conditions the seedlings experience diurnally, seasonally and among plots. Soil volumetric water content (VWC, %) was measured approximately weekly in treatment and control plots with a handheld electrical conductivity soil moisture probe inserted 5 cm vertically into the soil surface at three random locations within each droughted and ambient plot (HS2, Campbell Scientific, Logan, UT, USA). No seedlings (*n* = 82 fir and 62 spruce seedlings in drought treatments, 89 fir and 63 spruce seedlings in ambient conditions) died during the study.

### Morphological and physiological measurements

At the conclusion of the second growing season of precipitation exclusion (September 2019), a random subsample (*n* = 67, 14–18 individuals per species per treatment) of both species in each precipitation treatment was selected for gas exchange survey measurements. Net photosynthesis (*A*, μmol CO_2_·m^−2^·s^−1^), transpiration (*E*, mmol H_2_O·m^−2^·s^−1^), instantaneous water use efficiency (*A*/*E*) and stomatal conductance to water vapour (*g*_sw_, mol·m^−2^·s^−1^) were measured using a portable infrared gas analyser (LI-6800, LI-COR Biosciences, Lincoln, NE, USA). Sun-oriented sprigs of larger seedlings were inserted laterally in a 3 × 3 cm aperture chamber (LI 6800-12A), while entire leaf areas of smaller seedlings were inserted vertically via a modified gasket in the bottom of the same chamber, maintaining original sunwards seedling orientation. Chamber conditions were set to a saturating light intensity of 1200 μmol·m^−2^·s^−1^ photosynthetically active radiation using an LED light source (LI 6800-02) and 410 ppm CO_2_, representative of atmospheric CO_2_ levels reported nearby at the Niwot Ridge Global Monitoring Laboratory (CO, USA; NOAA ESRL 2020). Temperature inside the chamber was matched to ambient conditions every 10–15 min. Vapour pressure deficit averaged 2.0 (±0.3) for the duration of the sampling period. Measurements, averaged across 4 s, were logged once changes in CO_2_ and H_2_O concentrations had stabilized (<0.2 μmol·mol^−1^ and 0.1 mmol·mol^−1^ standard deviation for CO_2_ and H_2_O, respectively; ≈5 min per measurement). All measurements were conducted on 15–16 September 2019 under clear skies between 9:00 am and 1:00 pm. Measurements were corrected for silhouette leaf area of the total chamber leaf sample.

Following gas exchange measurements, tree seedling allometry was assessed by excavating seedlings from all plots, measuring stem diameter at the root crown to the nearest 0.01 mm to approximate seedling size and segregating each individual into component leaves, shoots and roots (*n* = 82 fir and 62 spruce seedlings in drought treatments, 89 fir and 63 spruce seedlings in ambient conditions). Total leaf area was determined via silhouette method using a flatbed scanner (Perfection V850, Epson, Nagano, Japan). Images captured at 600 DPI were thresholded and quantified for projected leaf area (cm^2^) using FIJI v. 1.52 (Schindelin *et al.* 2012). Leaf area for the purpose of gas exchange parameters was assessed in this same fashion. Leaf:stem area ratio (LSAR, cm^2^) as an approximation of leaf:sapwood area ratio was obtained by dividing leaf area by stem cross-sectional area calculated from stem diameter, assuming stems were circular in cross-section. Leaves, shoots and roots were then dried at 70 °C for 72 h and weighed. From these values, we calculated root, stem and leaf mass fraction (RMF, SMF and LMF, respectively, g·g^−1^) as a proportion of total biomass (TBM, g).

### Statistical analyses

Responses of allometry and gas exchange characteristics to treatments were modelled individually for each characteristic with mixed linear effect models. Seedling stem diameter (as a proxy for seedling age), species and treatment, as well as their interactions, were included as fixed effects, and plot was included as a random effect (package ‘nlme’; Pinheiro *et al.* 2021). For modelling, root, stem and leaf mass fractions were logit-transformed while total biomass and leaf-stem area ratios were log-transformed to improve normality of regression residuals. Plot-level transmitted light assessed with hemispherical photographs was at first also included to account for any light-availability effects on growth and physiology; however, no main or interactive effects of light were significant, and the term was therefore removed from all models. Fixed effects were evaluated on the basis of their unstandardized regression coefficients and significance at *α* = 0.05. Stem diameter-adjusted estimated marginal means were then calculated to directly compare the effect of treatment on mean allometry and gas exchange variables (package ‘emmeans’; Length 2021). Marginal means were estimated using the previous mixed linear effects models, but for each species separately such that treatment and diameter were the only fixed effects. Tukey *post hoc* testing was implemented to evaluate significant differences in the estimated means of allometry and gas exchange responses between control and drought treatments for each species separately. Means were back-transformed to their original scale for the purpose of comparison. All analyses were conducted in R v. 4.1 (R Core Team 2021).

## Results

### Soil moisture

**Figure 1.**
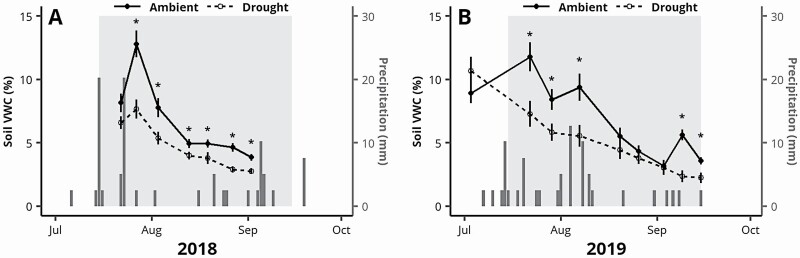
Mean soil volumetric water content (VWC, %) in ambient and drought treatments measured periodically in 2018 (A) and 2019 (B). Volumetric water content measurements began in 2018 shortly after the start of the drought treatments, and before treatments were started in 2019. The light grey area indicates dates which the drought treatment was active (15 July to 15 September). Daily precipitation (mm) is presented with dark grey bars (NRCS SNOTEL 2020). Error bars indicate standard error of the mean. Asterisks indicate significant difference in mean VWC between drought and ambient treatments for the given date (Tukey HSD, *P* < 0.05).

Relative to ambient conditions, precipitation exclusion shelters were successful at reducing soil water content *in situ* (Fig. 1). While no pre-treatment measurements were taken in 2018, significant soil dry-down in the drought treatments was evident within 2 weeks of the start of the precipitation exclusion treatment. Average soil moisture then remained significantly lower in the drought treatment for the remainder of the treatment period. Slight increases in VWC were observed immediately following larger precipitation events in the drought treatments (e.g. late July 2018), indicating some rain may have blown in laterally during windy precipitation events and/or subsurface water may have flowed into the plots from upslope drainage after saturating rains (Fig. 1A). In 2019, average soil water content did not significantly differ between ambient and droughted conditions prior to implementation of the treatment and followed a similar dry-down pattern to 2018. An exception occurred in late August and early September where low levels of precipitation resulted in soil drying within the ambient condition as well, resulting in non-significant treatments differences (Fig. 1B). Overall, droughted conditions remained on average 2.01 % (±0.55 SE) lower in soil VWC for the duration of precipitation exclusion treatment in 2018, and 2.17 % (±0.53 SE) lower in 2019, accounting for an estimated cumulative reduction of 125 % and 135 % soil VWC-days in 2018 and 2019, respectively.

### Morphological responses

Stem diameter was strongly predictive of all measures of tree allometry. Notably, root mass fraction was negatively correlated with diameter in both species where larger stem diameters were associated with a greater proportion of biomass allocation to above-ground tissues at the expense of the relative mass of roots **[see****Supporting Information—Table S2****]**. Species was also significantly predictive of root and leaf mass fractions—spruce consistently had lower root mass fractions than fir, and higher leaf mass fractions than fir. Species was however not predictive of stem mass fraction, total biomass, or leaf-stem area ratio. The effect of treatment was not significantly predictive of any measure of tree allometry or total biomass, nor were the interactions of diameter by treatment, species by treatment or diameter by species by treatment for most measures. Exceptions included the significant association of the interaction of diameter by treatment, species by treatment and diameter by species by treatment for stem mass fraction, and diameter by species for leaf mass fraction.

**Figure 2.**
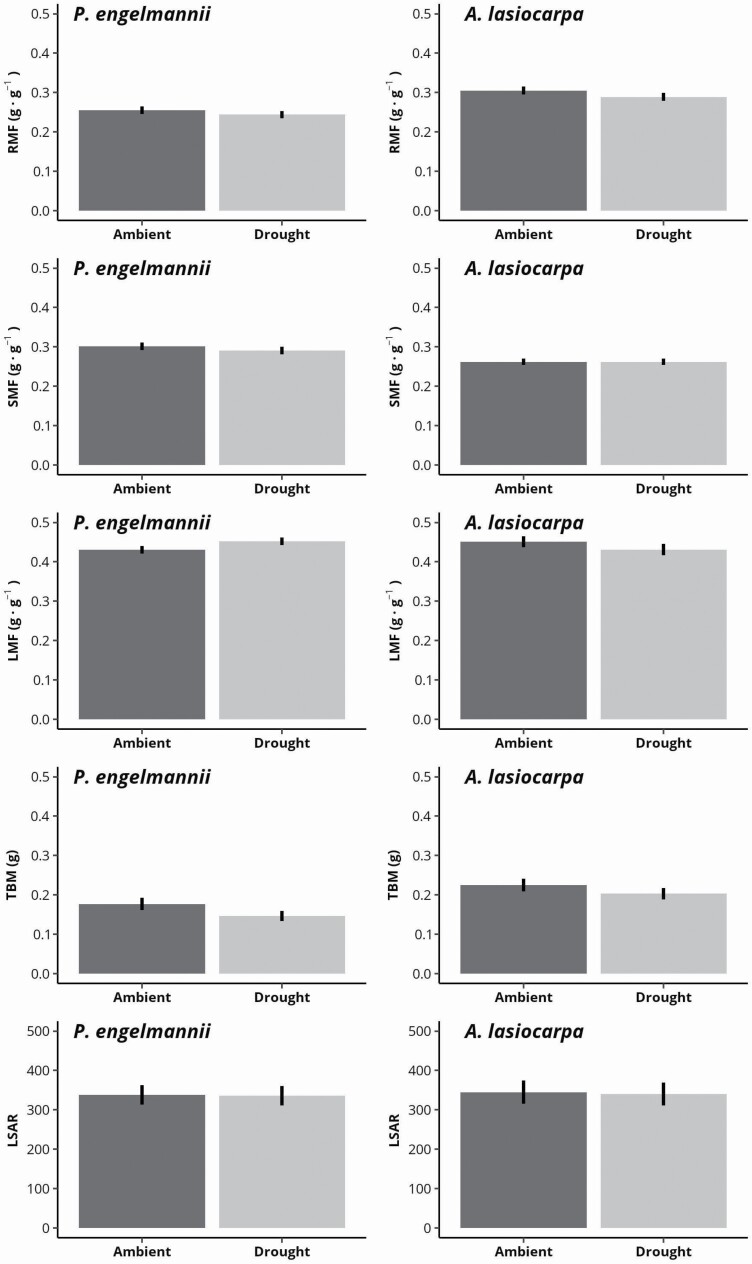
Estimated marginal means (mean) and standard errors (SE) of the effect of precipitation exclusion (drought vs. ambient) on root mass fraction (RMF, g·g^−1^), stem mass fraction (SMF, g·g^−1^), leaf mass fraction (LMF, g·g^−1^), total biomass (TBM, g) and leaf-stem area ratio (LSAR, cm^2^) of Engelmann spruce (*P. engelmannii*) and subalpine fir (*A. lasiocarpa*) tree seedlings. No significant differences between treatment means were found for either species (*P* > 0.05, Tukey HSD).

When controlling for stem diameter, treatment effects were not significant for any measure of allometry for either spruce or fir (Fig. 2). However, estimated marginal means of root mass fraction and total biomass were slightly lower in the drought treatment than the ambient treatment for both species (4.3 and 5.2 % lower RMF and 17.5 and 9.8 % lower TBM in drought conditions for spruce and fir, respectively), while leaf mass fraction averaged slightly higher in the drought treatment for spruce (4.9 %), and lower for fir (4.4 %). Leaf:stem area ratio remained similar (<1.5 % change) between treatments in both fir and spruce.

### Gas exchange responses

Among gas exchange parameters, only water use efficiency was significantly and positively associated with stem diameter **[see****Supporting Information—Table S3****]**. Stomatal conductance was greater in spruce. Net photosynthesis was negatively associated with treatment, though this effect was not significant. No gas exchange parameter was significantly associated with treatment, nor the interaction of diameter by species, diameter by treatment, species by treatment or diameter by species by treatment.

**Figure 3.**
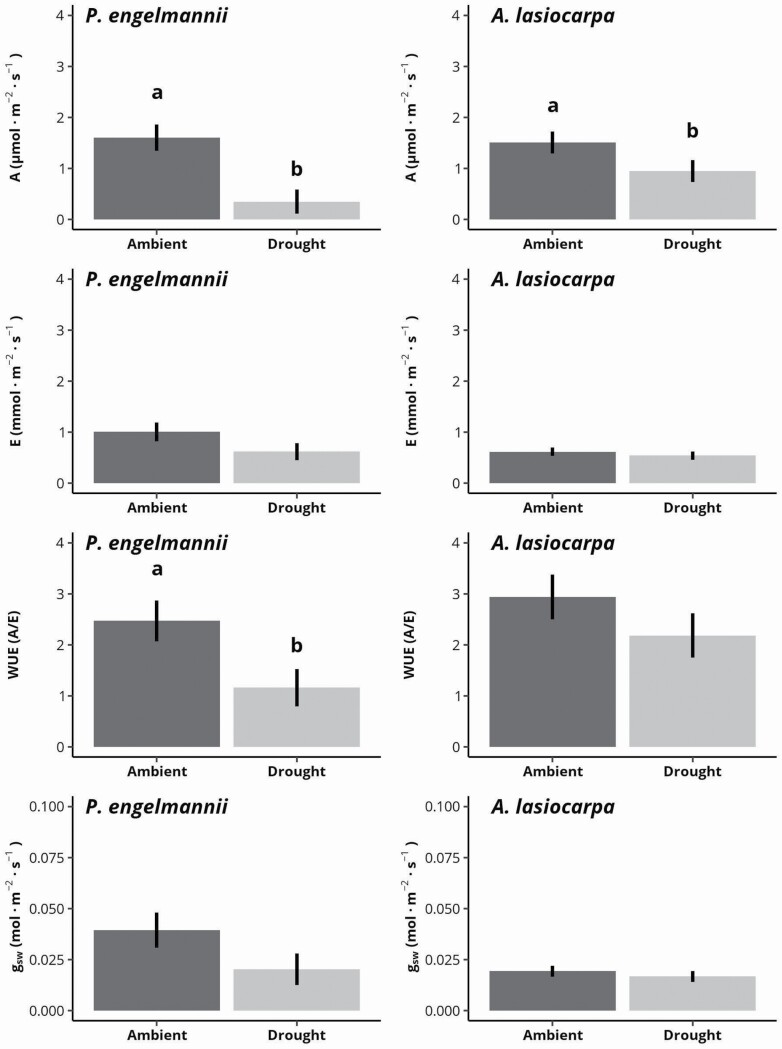
Estimated marginal means of net photosynthesis (*A*, μmol CO_2_·m^−2^·s^−1^), transpiration (*E*, mmol H_2_O·m^−2^·s^−1^), instantaneous water use efficiency (*A*/*E*) and stomatal conductance to water vapour (*g*_sw_, mol·m^−2^·s^−1^) for Engelmann spruce (*P. engelmannii*) and subalpine fir (*A. lasiocarpa*) tree seedlings subjected to ambient or drought conditions. Marginal means were adjusted for stem diameter. Error bars indicate standard error of the mean. Different letters indicate significant difference between means (Tukey HSD, *P* < 0.05).

Adjusted for stem diameter and evaluated individually for each species, estimated marginal means of numerous gas exchange properties were found to be substantially affected by the drought treatment (Fig. 3). In both fir and spruce, net photosynthesis was significantly lower for individuals in the drought treatment relative to those in ambient conditions, with a greater loss of net carbon gain exhibited by spruce (1.251 μmol CO_2_·m^−2^·s^−1^ [78.2 %, *P* = 0.046, Tukey HSD] mean reduction in spruce vs. 0.563 μmol CO_2_·m^−2^·s^−1^ [37.3 %, *P* = 0.001, Tukey HSD] mean reduction in fir). Transpiration was reduced, though not significantly, for both species (38.3 and 11.9 % in spruce and fir, *P* = 0.135 and 0.476, respectively, Tukey HSD). Instantaneous water use efficiency was significantly lower (53 %, *P* = 0.029, Tukey HSD) in spruce in response to the drought treatment, and while a loss of efficiency (25.9 %) was also found in fir, this effect was not significant (*P* = 0.127). Similarly, the reduction in stomatal conductance to water vapour, though insignificant, was greater in spruce than in fir (48.5 and 13.5 % in spruce and fir, *P* = 0.135 and 0.435, respectively, Tukey HSD).

## Discussion

Precipitation exclusion shelters were successful at substantially reducing *in situ* soil water compared to ambient conditions, particularly early in the treatment period when the droughted treatments averaged ≈5–8 % VWC and the ambient condition averaged ≈8–12 % VWC (Fig. 1). In the absence of water potential measurements, we are unable to unambiguously confirm inducement of plant water stress—reductions in soil VWC may not have amounted to meaningful reductions in physiological water availability, and seedlings may have accessed moisture pools deeper than the 5 cm soil horizon that we assessed for water content. However, in tree seedlings of other species, soil moisture levels <10 % VWC are often sufficient in meaningfully lowering stem water potentials, indicating our treatments were likely to induce considerable drought stress (Reinhardt *et al.* 2015; Kannenberg *et al.* 2019). Indeed, Lazarus *et al.* (2018) documented meaningful growth declines in conifer seedlings associated with just a mean 1 % reduction in soil VWC imposed with a heating treatment in a single growing season, indicating that even minor reductions in water availability can have considerable consequences for tree seedlings. The seedlings in the ambient condition in our study also likely experienced some degree of water stress in 2018 (15 July to 15 September) when the study region experienced a moderate drought (NDMG 2020)—this likely explains the near-convergence of soil water levels towards the end of the treatment period that season, though water levels remained significantly higher in the ambient condition for most of the 2018 measurements, indicating cumulative water stress was likely more severe in the drought treatment for the entire duration of study.

Measures of allometry—biomass fraction of roots, stem and leaves, total biomass and leaf:stem area ratio—were largely unaffected by 2 years of summer drought in both spruce and fir seedlings (**see****Supporting Information—Table S2**; Fig. 2). The lack of significant morphological responses contrasts with our expectation that seedlings would allocate more resources to root development to alleviate water stress or reduce leaf area to limit transpiration losses, and that these adjustments would be more apparent in spruce due to its relatively greater capacity for annual growth (Day 1964; Antos *et al.* 2000). Though the seedlings in our study were established, this behaviour could indicate larger individuals of these species continue to have little morphological responsiveness similar to first-year germinant seedlings which appear to prioritize leaf area gain over other structural traits, irrespective of water stress (e.g. Kiorapostolou *et al.* 2018; Augustine and Reinhardt 2019). However, we did find a significant negative correlation between stem diameter and proportional root mass, and a significant positive association to proportional stem and leaf biomass and leaf:stem area ratio, demonstrating that seedlings of both species regardless of drought treatment allocate an increasing proportion of resources to leaf development as they grow larger. In addition, we detected small, non-significant decreases in root mass and significant increases in leaf mass relative to total biomass in both species in response to drought, trends which could become important over longer periods if, as predicted, summer droughts become more frequent in the region. While mature trees are known to invest more heavily in support structures such as roots and branches as they age (Niinements 2010), our finding of greater leaf investment across a range of small seedling sizes may reflect a life-history strategy which emphasizes photosynthesis and growth rates over stress mitigation traits to increase the likelihood of the seedling successfully growing out of understory (Andrus *et al.* 2018). The short growing seasons in high-elevation ecosystems like the subalpine forests of the Rocky Mountain region can result in a strong selection of traits prioritizing carbon gain (Valladares *et al.* 2007, 2014; Vitasse *et al.* 2013; Gugger *et al.* 2015), a pattern which could explain the continued investment in leaf development across seedling sizes despite significant reductions in moisture, as observed in our study.

Given the extremely slow growth rates in these short growing seasons (Veblen 1986), it is also possible that significant morphological adjustments could occur in these species at timescales beyond the 2 years of our study and are likely trait-specific. For instance, McBranch *et al.* (2019) found no shift in leaf:sapwood area ratios in piñon pine (*Pinus edulis* Engelm.) and one-seed juniper (*Juniperus monosperma* (Engelm.) Sarg.) after 5 years of precipitation reduction and warming, but reductions in leaf and stem growth were found the year following implementation of the treatment in the same population (Adams *et al.* 2015). Further, size-related shifts in patterns of morphological acclimation in response to water deficit may occur after seedlings break from understory suppression and as saplings or small trees become subjected to changing microclimatic and resource conditions (Niinements 2010).

Though we found no significant morphological adjustments in response to summer drought, we observed species-specific and size-specific physiological changes between droughted and ambient-grown seedlings. In ambient conditions, spruce and fir displayed similar levels of photosynthesis, but under drought, spruce experienced a dramatic reduction in net photosynthesis (≈78 % reduction) compared to fir (≈37 % reduction; Fig. 3), supporting our hypothesis that fir maintains comparatively high levels of physiological functioning under water stress (Knapp and Smith 1981, 1982; Brodersen *et al.* 2006). In comparison, Gill *et al.* (2015) found photosynthetic flux was 42 % lower prior to water addition in droughted 3-year Engelmann spruce seedlings. Similarly, Broderson *et al.* (2006) noted a ≈50 % reduction in net photosynthesis in an adult population of both Engelmann spruce and subalpine fir during a summer drought relative to saplings sampled in the same region in prior wet years (Carter and Smith 1988), though differences in life stage and other environmental conditions between the studies make it uncertain if the reduction in photosynthesis was due exclusively to the drought.

Notably, neither species was able to effectively regulate stomatal conductance to increase water use efficiency in the face of persistent water deficits. In fact, we observed the opposite where little adjustment in stomatal conductance paired with non-proportional reductions in transpiration relative to photosynthesis resulted in significantly lower average instantaneous water use efficiencies in the drought treatments relative to ambient conditions, especially for spruce. However, we did find significantly greater water use efficiencies tied to increasing seedling size, suggesting greater responsivity to water stress with tree age **[see****Supporting Information—Table S3****]**, as found for 1- to 4-year-old subalpine fir seedlings (Cui and Smith 1991). The lack of a morphological response to reduced water levels by the seedlings in this study further suggests carbon gain is strongly prioritized in small seedlings until certain developmental levels are met, such as thresholds in age, size, allometric ratio or external conditions (e.g. release from low light levels in the understory). Once such a threshold is reached, individuals may begin to display greater morphological and physiological responsivity to environment stress, as has been observed for saplings and adults of these species (Knapp and Smith 1981; Brodersen *et al.* 2006). While our study, through the investigation of a broad range of seedling sizes reflective of a naturally regenerating seedling bank (ca. 300 seedlings < 5 mm in basal diameter), has provided preliminary evidence on such canalization of stress insensitivity in seedlings well after establishment, assessments along a finer ontogenetic gradient could help resolve uncertainty in the timing and mechanisms by which seedlings develop stress responses more similar to adult conspecifics.

In the subalpine forests of the southern Rocky Mountains, late-successional co-dominance between subalpine fir and Engelmann spruce is maintained by contrasting life-history traits between species. Fir is more dominant in the shady understories of mature forests, substantially outnumbering spruce in the seedling bank. However, spruce is more successful at recruiting from the seedling bank into canopy gaps due to faster growth, and with its greater longevity, this allows spruce to remain co-dominant in the overstory (Andrus *et al.* 2018). Our results show that at the end of two growing seasons of precipitation reduction fir was far less affected physiologically—with much smaller reductions in net photosynthesis and water use. These results suggest the favourability of spruce in higher light could be reduced by its poorer physiological functioning under water deficits. If periods of increasing drought frequency and severity were to exceed its tolerance, spruce may face greater physiological regeneration barriers than fir, potentially leading to disruption of the delicate regeneration dynamics between the two species, with long-term implications for forest structure and function.

## Conclusion

Knowledge of species’ ability to acclimate to shifts in precipitation regimes at the seedling stage will provide a vital understanding of the underlying traits that convey drought tolerance. This understanding will further illuminate how these capacities vary within and among species occupying a critical regeneration bottleneck in high-elevation forest systems. In this study we demonstrate a lack of morphological and physiological responses to consecutive summer drought in seedlings of the two dominant subalpine species of the southern Rocky Mountains—Engelmann spruce and subalpine fir. No morphological adjustments to drought mitigation traits were detected in either species, and both photosynthetic carbon gain and water use efficiency were greatly reduced reflecting poor whole-seedling acclimation to water stress, particularly for spruce. Further, increases in above-ground biomass allocation in response to seedling size did not reflect expected shifts towards greater investment in structural traits over carbon gain structures as seedlings grew in size. However, while no increase in morphological investment was observed, increasing stomatal control with seedling size conferred greater water use efficiency in larger individuals. These results suggest strong conservation of traits that support short-term carbon gain at the expense of water stress mitigation well into understory establishment. Increased seedling mortality with climate change-induced drought is a likely outcome of these responses, which may in turn affect availability of seedlings for recruitment into larger tree size classes, thus disrupting the regeneration dynamics that maintain species co-dominance.

## Supporting Information

The following additional information is available in the online version of this article—

**Table S1.** Monthly average temperature (°C), total precipitation (mm) and average snow depth (cm) for years preceding (2017) and during (2018–19) study at the field site near Mt. Evans, CO, USA.

**Table S2.** Linear mixed-effects model estimates (*B*, unstandardized coefficients, SE, standard errors, df, degrees of freedom, *T* and *P*-value) of root mass fraction (RMF, g·g^−1^), stem mass fraction (SMF, g·g^−1^), leaf mass fraction (LMF, g·g^−1^), total biomass (TBM, g) and leaf-stem area ratio (LSAR, cm^2^) modelled individually as a function of diameter, species and drought treatment as fixed effects and plot as a random effect for Engelmann seedlings.

**Table S3.** Linear mixed-effects model estimates (*B*, unstandardized coefficients, SE, standard errors, df, degrees of freedom, *T* and *P*-value) of net photosynthesis (*A*, μmol CO^2^·m^−2^·s^−1^), transpiration (*E*, mmol H_2_O·m^−2^·s^−1^), instantaneous water use efficiency (*A*/*E*) and stomatal conductance to water vapour (*g*_sw_, mol·m^−2^·s^−1^) modelled individually as a function of diameter, species and drought treatment as fixed effects and plot as a random effect for Engelmann spruce (*P. engelmannii*) and subalpine fir (*A. lasiocarpa*) tree seedlings.

**Figure S1.** Distribution of Engelmann spruce (*P. engelmannii*, *n* = 125) and subalpine fir (*A. lasiocarpa*, *n* = 171) tree seedling sizes (stem diameter, mm) subjected to ambient and drought conditions.

plab077_suppl_Supplementary_Material

## Data Availability

The data that support the findings of this study are openly available on GitHub at: https://github.com/atgoke/ManuscriptData.

## Notes

**Plants, Ecosystems & Climate. Chief Editor**: Mary Heskel

## References

[CIT0001] Adams HD , Collins AD, Briggs SP, Vennetier M, Dickman LT, Sevanto SA, Garcia-Forner N, Powers HH, McDowell NG. 2015. Experimental drought and heat can delay phenological development and reduce foliar and shoot growth in semiarid trees. Global Change Biology 21:4210–4220. 26149972 10.1111/gcb.13030

[CIT0002] Allen CD , Macalady AK, Chenchouni H, Bachelet D, McDowell N, Vennetier M, Kitzberger T, Rigling A, Breshears D, Hogg EH, Gonzalez P, Fensham R, Zhang Z, Castro J, Demidova N, Lim J, Allard G, Running S, Semerci A, Cobb N. 2010. A global overview of drought and heat-induced tree mortality reveals emerging climate change risks for forests. Forest Ecology and Management 259:660–684.

[CIT0003] Anderegg WR , Kane JM, Anderegg LD. 2013. Consequences of widespread tree mortality triggered by drought and temperature stress. Nature Climate Change 3:30–36.

[CIT0004] Andrus RA , Chai RK, Harvey BJ, Rodman KC, Veblen TT. 2021. Increasing rates of subalpine tree mortality linked to warmer and drier summers. Journal of Ecology 109:2203–2218.

[CIT0005] Andrus RA , Harvey BJ, Chai RK, Veblen TT. 2018. Different vital rates of Engelmann spruce and subalpine fir explain discordance in understory and overstory dominance. Canadian Journal of Forest Research 48:1554–1562.

[CIT0006] Antos JA , Parish R, Conley K. 2000. Age structure and growth of the tree-seedling bank in subalpine spruce-fir forests of south-central British Columbia. The American Midland Naturalist 143:342–354.

[CIT0007] Aranda I , Alía R, Ortega U, Dantas ÂK, Majada J. 2010. Intra-specific variability in biomass partitioning and carbon isotopic discrimination under moderate drought stress in seedlings from four *Pinus pinaster* populations. Tree Genetics & Genomes 6:169–178.

[CIT0008] Augustine SP , Reinhardt K. 2019. Differences in morphological and physiological plasticity in two species of first-year conifer seedlings exposed to drought result in distinct survivorship patterns. Tree Physiology 39:1446–1460. 31181151 10.1093/treephys/tpz048

[CIT0009] Bansal S , Germino MJ. 2008. Carbon balance of conifer seedlings at timberline: relative changes in uptake, storage, and utilization. Oecologia 158:217–227. 18810499 10.1007/s00442-008-1145-4

[CIT0010] Bansal S , Germino MJ. 2010. Variation in ecophysiological properties among conifers at an ecotonal boundary: comparison of establishing seedlings and established adults at timberline. Journal of Vegetation Science 21:133–142.

[CIT0011] Bell DM , Bradford JB, Lauenroth WK. 2014. Early indicators of change: divergent climate envelopes between tree life stages imply range shifts in the western United States. Global Ecology and Biogeography 23:168–180.

[CIT0012] Beniston M , Diaz HF, Bradley RS. 1997. Climatic change at high elevation sites: an overview. Climatic Change 36:233–251.

[CIT0013] Bigler C , Gavin DG, Gunning C, Veblen TT. 2007. Drought induces lagged tree mortality in a subalpine forest in the Rocky Mountains. Oikos 116:1983–1994.

[CIT0014] Brodersen CR , Germino MJ, Johnson DM, Reinhardt K, Smith WK, Resler LM, Bader M, Sala A, Kueppers L, Broll G, Cairns D, Holtmeier FK, Weiser G. 2019. Seedling survival at timberline is critical to conifer mountain forest elevation and extent. Frontiers in Forests and Global Change 2:9.

[CIT0015] Brodersen CR , Germino MJ, Smith WK. 2006. Photosynthesis during an episodic drought in *Abies lasiocarpa* and *Picea engelmannii* across an alpine treeline. Arctic, Antarctic, and Alpine Research 38:34–41.

[CIT0016] Buechling A , Martin PH, Canham CD. 2017. Climate and competition effects on tree growth in Rocky Mountain forests. Journal of Ecology 105:1636–1647.

[CIT0017] Carroll CJW , Knapp AK, Martin PH. 2017. Dominant tree species of the Colorado Rockies have divergent physiological and morphological responses to warming. Forest Ecology and Management 402:234–240.

[CIT0018] Carroll CJW , Knapp AK, Martin PH. 2021. Higher temperatures increase growth rates of Rocky Mountain montane tree seedlings. Ecosphere 12:e03414.

[CIT0019] Carter GA , Smith WK. 1988. Microhabitat comparisons of transpiration and photosynthesis in three subalpine conifers. Canadian Journal of Botany 66:963–969.

[CIT0020] Cherwin K , Knapp A. 2012. Unexpected patterns of sensitivity to drought in three semi-arid grasslands. Oecologia 169:845–852. 22223080 10.1007/s00442-011-2235-2

[CIT0021] Choat B , Brodribb TJ, Brodersen CR, Duursma RA, López R, Medlyn BE. 2018. Triggers of tree mortality under drought. Nature 558:531–539. 29950621 10.1038/s41586-018-0240-x

[CIT0022] Clark JS , Iverson L, Woodall CW, Allen CD, Bell DM, Bragg DC, D’Amato AW, Davis FW, Hersh MH, Ibanez I, Jackson ST, Matthews S, Pederson N, Peters M, Schwartz MW, Waring KM, Zimmermann NE. 2016. The impacts of increasing drought on forest dynamics, structure, and biodiversity in the United States. Global Change Biology 22:2329–2352. 26898361 10.1111/gcb.13160

[CIT0023] Conlisk E , Castanha C, Germino MJ, Veblen TT, Smith JM, Moyes AB, Kueppers LM. 2018. Seed origin and warming constrain lodgepole pine recruitment, slowing the pace of population range shifts. Global Change Biology 24:197–211. 28746786 10.1111/gcb.13840

[CIT0024] Cook BI , Seager R. 2013. The response of the North American monsoon to increased greenhouse gas forcing. Journal of Geophysical Research: Atmosphere 118:1690–1699.

[CIT0025] Copenhaver-Parry PE , Carroll CJW, Martin PH, Talluto MV. 2020. Multi-scale integration of tree recruitment and range dynamics in a changing climate. Global Ecology and Biogeography 29:102–116.

[CIT0026] Cui M , Smith WK. 1991. Photosynthesis, water relations and mortality in *Abies lasiocarpa* seedlings during natural establishment. Tree Physiology 8:37–46. 14972895 10.1093/treephys/8.1.37

[CIT0027] Day RJ . 1964. The microenvironments occupied by spruce and fir regeneration in the Rocky Mountains. Publication 1037. Ottawa, Canada: Forest Research Branch, Canadian Department of Forestry.

[CIT0028] Dobrowski SZ , Parks SA. 2016. Climate change velocity underestimates climate change exposure in mountainous regions. Nature Communications 7:12349. 10.1038/ncomms12349 PMC4974646 27476545

[CIT0029] Drought-Net . 2018. Drought-Net research coordination network. Colorado State University. https://drought-net.colostate.edu/ (15 May 2018).

[CIT0030] Fay PA , Carlisle JD, Knapp AK, Blair JM, Collins SL. 2000. Altering rainfall timing and quantity in a mesic grassland ecosystem: design and performance of rainfall manipulation shelters. Ecosystems 3:308–319.

[CIT0031] Foster AC , Martin PH, Redmond MD. 2020. Soil moisture strongly limits Douglas-fir seedling establishment near its upper elevational limit in the southern Rocky Mountains. Canadian Journal of Forest Research 50:837–842.

[CIT0032] Frazer GW , Canham CD, Lertzman KP. 1999. Gap light analyzer (GLA): imaging software to extract canopy structure and gap light transmission indices from true-colour fisheye photographs. British Columbia, Canada: Simon Fraser University and New York: The Institute of Ecosystem Studies.

[CIT0033] Gill RA , Campbell CS, Karlinsey SM. 2015. Soil moisture controls Engelmann spruce (*Picea engelmannii*) seedling carbon balance and survivorship at timberline in Utah, USA. Canadian Journal of Forest Research 45:1845–1852.

[CIT0034] Green DS . 2005. Adaptive strategies in seedlings of three co-occurring, ecologically distinct northern coniferous tree species across an elevational gradient. Canadian Journal of Forest Research 35:910–917.

[CIT0035] Grossnickle SC . 2005. Importance of root growth in overcoming planting stress. New Forests 30:273–294.

[CIT0036] Gugger S , Kesselring H, Stöcklin J, Hamann E. 2015. Lower plasticity exhibited by high- versus mid-elevation species in their phenological responses to manipulated temperature and drought. Annals of Botany 116:953–962. 26424784 10.1093/aob/mcv155 PMC4640129

[CIT0037] Heisler-White JL , Knapp AK, Kelly EF. 2008. Increasing precipitation event size increases aboveground net primary productivity in a semi-arid grassland. Oecologia 158:129–140. 18670792 10.1007/s00442-008-1116-9

[CIT0038] Hu JIA , Moore DJ, Burns SP, Monson RK. 2010. Longer growing seasons lead to less carbon sequestration by a subalpine forest. Global Change Biology 16:771–783.

[CIT0039] Intergovernmental Panel on Climate Change . 2014. Climate change 2014: impacts, adaptation, and vulnerability. Working Group II Contribution to the IPCC 5th Assessment Report. Stanford, CA: Intergovernmental Panel on Climate Change.

[CIT0040] Kannenberg SA , Novick KA, Phillips RP. 2019. Anisohydric behavior linked to persistent hydraulic damage and delayed drought recovery across seven North American tree species. The New Phytologist 222:1862–1872. 30664253 10.1111/nph.15699

[CIT0041] Kiorapostolou N , Galiano-Pérez L, von Arx G, Gessler A, Petit G. 2018. Structural and anatomical responses of *Pinus sylvestris* and *Tilia platyphyllos* seedlings exposed to water shortage. Trees 32:1211–1218.

[CIT0042] Knapp AK , Smith WK. 1981. Water relations and succession in subalpine conifers in southeastern Wyoming. Botanical Gazette 142:502–511.

[CIT0043] Knapp AK , Smith WK. 1982. Factors influencing understory seedling establishment of Engelmann spruce (*Picea engelmannii*) and subalpine fir (*Abies lasiocarpa*) in southeast Wyoming. Canadian Journal of Botany 60:2753–2761.

[CIT0044] Lazarus BE , Castanha C, Germino MJ, Kueppers LM, Moyes AB. 2018. Growth strategies and threshold responses to water deficit modulate effects of warming on tree seedlings from forest to alpine. Journal of Ecology 106:571–585.

[CIT0045] Length R . 2021. emmeans: estimated marginal means, aka least-squares means. R package version 1.6.2-1. https://CRAN.R-project.org/package=emmeans (10 July 2021).

[CIT0046] Lett S , Dorrepaal E. 2018. Global drivers of tree seedling establishment at alpine treelines in a changing climate. Functional Ecology 32:1666–1680.

[CIT0047] Lukas J , Barsugli J, Doesken N, Rangwala I, Wolter K. 2014. Climate change in Colorado: a synthesis to support water resources management and adaptation. Boulder, CO: University of Colorado.

[CIT0048] Martin PH , Canham CD. 2020. Peaks in frequency, but not relative abundance, occur in the center of tree species distributions on climate gradients. Ecosphere 11: e03149.

[CIT0049] Martínez-Vilalta J , Lloret F. 2016. Drought-induced vegetation shifts in terrestrial ecosystems: the key role of regeneration dynamics. Global and Planetary Change 144:94–108.

[CIT0050] McBranch NA , Grossiord C, Adams H, Borrego I, Collins AD, Dickman T, Ryan M, Sevanto S, McDowell NG. 2019. Lack of acclimation of leaf area:sapwood area ratios in piñon pine and juniper in response to precipitation reduction and warming. Tree Physiology 39:135–142. 30272223 10.1093/treephys/tpy066

[CIT0051] Mote PW , Hamlet AF, Clark MP, Lettenmaier DP. 2005. Declining mountain snowpack in western North America. Bulletin of the American Meteorological Society 86:39–50.

[CIT0052] NDMG . 2020. U.S. drought monitor. National Drought Mitigation Center. https://droughtmonitor.unl.edu/Data.aspx (26 August 2020).

[CIT0053] Niinemets Ü . 2010. Responses of forest trees to single and multiple environmental stresses from seedlings to mature plants: past stress history, stress interactions, tolerance and acclimation. Forest Ecology and Management 260:1623–1639.

[CIT0054] NOAA ESRL . 2020. Global monitoring laboratory GML data finder. Earth System Research Laboratories, National Oceanic and Atmospheric Association. https://www.esrl.noaa.gov/gmd/dv/data/index.php (26 August 2020).

[CIT0055] NRCS SNOTEL . 2020. Snow telemetry (SNOTEL) and snow course data and products. Natural Resources Conservation Service, United States Department of Agriculture. https://wcc.sc.egov.usda.gov/nwcc/site?sitenum=936 (20 May 2020).

[CIT0056] NRCS . 2020. Web Soil Survey. Natural Resources Conservation Service, United States Department of Agriculture. Available from: https://websoilsurvey.sc.egov.usda.gov/App/HomePage.htm (20 May 2020).

[CIT0057] Pascale S , Boos WR, Bordoni S, Delworth TL, Kapnick SB, Murakami H, Vecchi G, Zhang W. 2017. Weakening of the North American monsoon with global warming. Nature Climate Change 7:806–812.

[CIT0058] Pepin N , Bradley RS, Diaz HF, Baraër M, Caceres EB, Forsythe N, Fowler H, Greenwood G, Hashmi MZ, Liu XD, Miller JR, Ning L, Ohmura A, Palazzi E, Rangwala I, Schoner W, Severskiy I, Shahgedanova M, Wang MB, Williamson SN, Yang DQ. 2015. Elevation-dependent warming in mountain regions of the world. Nature Climate Change 5:424–430.

[CIT0059] Pinheiro J , Bates D, DebRoy S, Sarkar D, R Core Team. 2021. *nlme: linear and nonlinear mixed effects models*. *R package version 3.1-153*. http://CRAN.R-project.org/package=nlme (20 September 2021).

[CIT0060] R Core Team . 2021. R: a language and environment for statistical computing. Vienna, Austria: R Foundation for Statistical Computing. https://www.R-project.org/ (10 July 2021).

[CIT0061] Reinhardt K , Germino MJ, Kueppers LM, Domec JC, Mitton J. 2015. Linking carbon and water relations to drought-induced mortality in *Pinus flexilis* seedlings. Tree Physiology 35:771–782. 26116925 10.1093/treephys/tpv045

[CIT0062] Richter S , Kipfer T, Wohlgemuth T, Calderón Guerrero C, Ghazoul J, Moser B. 2012. Phenotypic plasticity facilitates resistance to climate change in a highly variable environment. Oecologia 169:269–279. 22081261 10.1007/s00442-011-2191-x

[CIT0073] Salley SW , Sleezer RO, Bergstrom RM, Martin PH, Kelly EF. 2016. A long-term analysis of the historical dry boundary for the Great Plains of North America: implications of climatic variability and climatic change on temporal and spatial patterns in soil moisture. Geoderma 274:104–113.

[CIT0063] Schall P , Lödige C, Beck M, Ammer C. 2012. Biomass allocation to roots and shoots is more sensitive to shade and drought in European beech than in Norway spruce seedlings. Forest Ecology and Management 266:246–253.

[CIT0064] Schindelin J , Arganda-Carreras I, Frise E, Kaynig V, Longair M, Pietzsch T, Preibisch S, Rueden C, Saalfeld S, Schmid B, Tinevez JY, White DJ, Hartenstein V, Eliceiri K, Tomancak P, Cardona A. 2012. Fiji: an open-source platform for biological-image analysis. Nature Methods 9:676–682. 22743772 10.1038/nmeth.2019 PMC3855844

[CIT0065] Shea KL . 1985. Demographic aspects of coexistence in Engelmann spruce and subalpine fir. American Journal of Botany 72:1823–1833.

[CIT0066] Smith JM , Paritsis J, Veblen TT, Chapman TB. 2015. Permanent forest plots show accelerating tree mortality in subalpine forests of the Colorado Front Range from 1982 to 2013. Forest Ecology and Management 341:8–17.

[CIT0067] Valladares F , Gianoli E, Gómez JM. 2007. Ecological limits to plant phenotypic plasticity. The New Phytologist 176:749–763. 17997761 10.1111/j.1469-8137.2007.02275.x

[CIT0068] Valladares F , Matesanz S, Guilhaumon F, Araújo MB, Balaguer L, Benito-Garzón M, Cornwell W, Gianoli E, van Kleunen M, Naya DE, Nicotra AB, Poorter H, Zavala MA. 2014. The effects of phenotypic plasticity and local adaptation on forecasts of species range shifts under climate change. Ecology Letters 17:1351–1364. 25205436 10.1111/ele.12348

[CIT0069] Van Mantgem PJ , Stephenson NL, Byrne JC, Daniels LD, Franklin JF, Fulé PZ, Harmon ME, Larson AJ, Smith JM, Taylor AH, Veblen TT. 2009. Widespread increase of tree mortality rates in the western United States. Science 323:521–524. 19164752 10.1126/science.1165000

[CIT0070] Veblen TT . 1986. Age and size structure of subalpine forests in the Colorado Front Range. Bulletin of the Torrey Botanical Club 113:225–240.

[CIT0071] Vitasse Y , Hoch G, Randin CF, Lenz A, Kollas C, Scheepens JF, Körner C. 2013. Elevational adaptation and plasticity in seedling phenology of temperate deciduous tree species. Oecologia 171:663–678. 23306445 10.1007/s00442-012-2580-9

[CIT0072] Yahdjian L , Sala OE. 2002. A rainout shelter design for intercepting different amounts of rainfall. Oecologia 133:95–101. 28547315 10.1007/s00442-002-1024-3

