# Supplementary material for: Poor acclimation to experimental field drought in subalpine forest tree seedlings
Source: AoB Plants. 2021 Dec 17;14(1):plab077. doi: 10.1093/aobpla/plab077 (PMC8782599; doi:10.1093/aobpla/plab077)
Supplement: plab077_suppl_Supplementary_Material [file plab077_suppl_supplementary_material.pdf]

Table S1: Monthly average temperature (°C), total precipitation (mm), and average snow depth (cm) for years preceding (2017) and during (2018-2019) study at the field site near Mt. Evans, CO, USA. Mean values are also presented from a reference period of 1999-2019 (NRCS SNOTEL 2020).

Site Characteristic	Year(s)	Jan	Feb	Mar	Apr	May	Jun	Jul	Aug	Sep	Oct	Nov	Dec
**Temperature**	**2017**	-6.8	-2.2	1.2	0.7	4.3	11.2	12.6	10.9	8.3	3.1	1.6	-2.8
	**2018**	-2.6	-5.3	-2.1	1.3	6.9	12.3	13.3	12.2	10.3	1.8	-4.2	-6.4
	**2019**	-5.6	-6.5	-3.4	1.9	2.3	8.8	13.2	13.1	10.9	0	-1.3	-5
	**Mean**	-5.3	-5.5	-2.1	0.8	4.7	10.3	12.9	11.5	8.5	3.1	-1.8	-6.0
**Precipitation**	**2017**	70.9	22.9	86.2	86.3	121.5	15.2	106.4	63.1	60.9	58.3	25.3	30.2
	**2018**	35.2	27.7	48.0	45.4	55.7	20.2	55.7	15.1	30.4	45.4	32.6	12.6
	**2019**	60.6	32.8	98.8	60.8	109.1	45.5	37.8	40.6	10.0	53.3	37.9	37.8
	**Mean**	34.9	39.0	66.2	96.8	78.4	38.8	89.4	72.5	50.7	51.0	32.3	37.3
**Snow Depth**	**2017**	49.8	52.8	56.5	77.2	56.7	3.2	0.0	0.0	0.6	9.6	10.3	12.8
	**2018**	13.4	26.9	30.8	31.8	6.5	0.0	0.0	0.0	0.0	7.6	23.2	27.5
	**2019**	48.8	55.7	87.5	75.3	56.9	7.4	0.0	0.0	0.0	12.3	30.5	49.5
	**Mean**	38.7	49.7	65.2	68.4	43.9	4.6	1.3	0.3	1.0	8.6	19.3	35.6

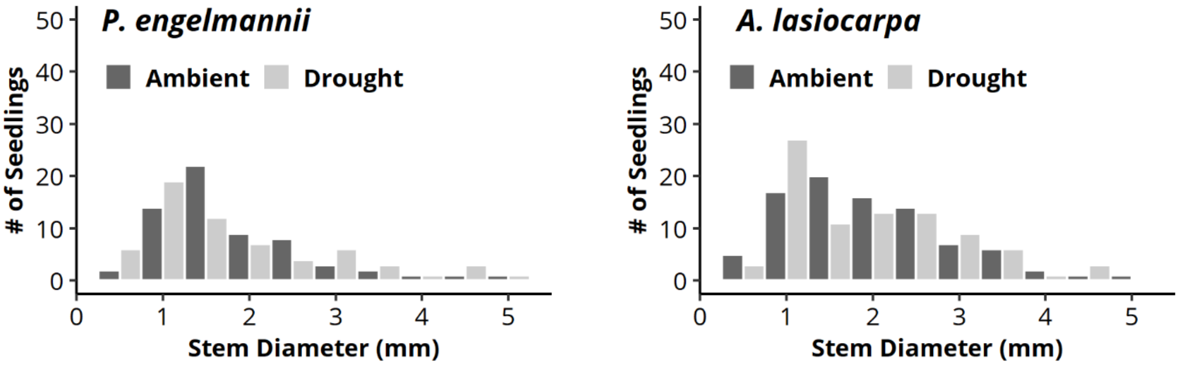

Figure S1: Distribution of Engelmann spruce (*P. engelmannii*, n=125) and subalpine fir (*A. lasiocarpa*, n=171) tree seedling sizes (stem diameter, mm) subjected to ambient and drought conditions.

Table S2: Linear mixed-effects model estimates (*B*, unstandardized coefficients, SE, standard errors, df, degrees of freedom, T, and p-value) of root mass fraction (RMF, $g \cdot g^{-1}$), stem mass fraction (SMF, $g \cdot g^{-1}$), leaf mass fraction (LMF, $g \cdot g^{-1}$), total biomass (TBM, g), and leaf-stem area ratio (LSAR, cm^2) modelled individually as a function of diameter, species, and drought treatment as fixed effects and plot as a random effect for Engelmann spruce (*P. engelmannii*) and subalpine fir (*A. lasiocarpa*) tree seedlings. Significant p-values (α = 0.05) are bolded.

Fixed Effects	*B*	SE	df	T	*p*
**RMF**					
**Diameter**	-0.28	0.04	273	-6.54	**< 0.001**
**Species (Spruce)**	-0.38	0.14	273	-2.74	**0.007**
**Treatment (Drought)**	0.02	0.12	273	0.14	0.887
**Diameter x Species**	0.05	0.07	273	0.73	0.464
**Diameter x Treatment**	-0.05	0.06	273	-0.82	0.412
**Species x Treatment**	-0.22	0.18	273	-1.24	0.217
**Diameter x Species x Treatment**	0.12	0.08	273	1.46	0.145
**SMF**					
**Diameter**	0.14	0.04	273	3.58	**< 0.001**
**Species (Spruce)**	0.12	0.13	273	0.96	0.340
**Treatment (Drought)**	-0.22	0.12	273	-1.91	0.057
**Diameter x Species**	0.06	0.06	273	1.01	0.315
**Diameter x Treatment**	0.11	0.05	273	2.07	**0.039**
**Species x Treatment**	0.35	0.17	273	2.07	**0.039**
**Diameter x Species x Treatment**	-0.21	0.08	273	-2.60	**0.010**
**LMF**					
**Diameter**	0.14	0.04	273	3.31	**0.001**
**Species (Spruce)**	0.31	0.14	273	2.21	**0.028**
**Treatment (Drought)**	0.19	0.12	273	1.54	0.125
**Diameter x Species**	-0.14	0.07	273	-2.06	**0.041**
**Diameter x Treatment**	-0.06	0.06	273	-1.13	0.261
**Species x Treatment**	-0.18	0.18	273	-0.96	0.335
**Diameter x Species x Treatment**	0.10	0.09	273	1.19	0.237
**TBM**					
**Diameter**	1.37	0.07	273	20.80	**< 0.001**
**Species (Spruce)**	-0.04	0.22	273	-0.19	0.846
**Treatment (Drought)**	-0.09	0.19	273	-0.45	0.650
**Diameter x Species**	-0.02	0.11	273	-0.15	0.885
**Diameter x Treatment**	-0.01	0.09	273	-0.08	0.936
**Species x Treatment**	-0.31	0.28	273	-1.09	0.279
**Diameter x Species x Treatment**	0.12	0.13	273	0.89	0.377
**LSAR**					
**Diameter**	0.37	0.07	273	5.65	**< 0.001**
**Species (Spruce)**	0.23	0.22	273	1.06	0.288
**Treatment (Drought)**	0.13	0.19	273	0.66	0.507
**Diameter x Species**	-0.11	0.11	273	-1.08	0.283
**Diameter x Treatment**	-0.07	0.09	273	-0.82	0.411
**Species x Treatment**	-0.37	0.28	273	-1.33	0.186
**Diameter x Species x Treatment**	0.20	0.13	273	1.52	0.129

Table S3: Linear mixed-effects model estimates (*B*, unstandardized coefficients, SE, standard errors, df, degrees of freedom, T, and p-value) of net photosynthesis (A, µmol $CO^2 \cdot m^{-2} \cdot s^{-1}$), transpiration (E, mmol $H_2O \cdot m^{-2} \cdot s^{-1}$), instantaneous water use efficiency (A/E), and stomatal conductance to water vapor (g_{sw}, mol $\cdot m^{-2} \cdot s^{-1}$) modelled individually as a function of diameter, species, and drought treatment as fixed effects and plot as a random effect for Engelmann spruce (*P. engelmannii*) and subalpine fir (*A. lasiocarpa*) tree seedlings. Significant p-values (α = 0.05) are bolded.

Fixed Effects	*B*	SE	df	T	*p*
**A**					
**Diameter**	0.13	0.20	44	0.67	0.507
**Species (Spruce)**	0.13	0.69	44	0.18	0.854
**Treatment (Drought)**	-0.29	0.65	44	-0.44	0.660
**Diameter x Species**	-0.03	0.31	44	-0.09	0.929
**Diameter x Treatment**	-0.14	0.27	44	-0.50	0.620
**Species x Treatment**	-1.63	0.94	44	-1.73	0.091
**Diameter x Species x Treatment**	0.51	0.43	44	1.17	0.248
**E**					
**Diameter**	-0.14	0.11	44	-1.26	0.215
**Species (Spruce)**	0.77	0.40	44	1.93	0.060
**Treatment (Drought)**	-0.00	0.37	44	-0.01	0.996
**Diameter x Species**	-0.24	0.18	44	-1.33	0.189
**Diameter x Treatment**	-0.04	0.16	44	-0.23	0.823
**Species x Treatment**	-0.77	0.54	44	-1.43	0.160
**Diameter x Species x Treatment**	0.25	0.25	44	1.01	0.317
**WUE**					
**Diameter**	0.80	0.34	44	2.37	**0.022**
**Species (Spruce)**	-0.99	1.19	44	-0.83	0.412
**Treatment (Drought)**	0.11	1.10	44	0.10	0.923
**Diameter x Species**	0.36	0.55	44	0.65	0.517
**Diameter x Treatment**	-0.41	0.46	44	-0.90	0.372
**Species x Treatment**	-0.63	1.60	44	-0.39	0.695
**Diameter x Species x Treatment**	-0.02	0.73	44	-0.03	0.975
g_{sw}					
**Diameter**	0.00	0.00	44	-1.00	0.324
**Species (Spruce)**	0.04	0.02	44	2.14	**0.038**
**Treatment (Drought)**	0.00	0.02	44	-0.03	0.978
**Diameter x Species**	-0.01	0.01	44	-1.32	0.193
**Diameter x Treatment**	0.00	0.01	44	-0.15	0.884
**Species x Treatment**	-0.04	0.02	44	-1.58	0.120
**Diameter x Species x Treatment**	0.01	0.01	44	1.05	0.300
